# A new nomogram model for prognosis of hepatocellular carcinoma based on novel gene signature that regulates cross-talk between immune and tumor cells

**DOI:** 10.1186/s12885-022-09465-9

**Published:** 2022-04-09

**Authors:** Youpeng Wang, Yeni Yang, Ziyin Zhao, Hongfa Sun, Dingan Luo, Lakshmi Huttad, Bingyuan Zhang, Bing Han

**Affiliations:** 1 Department of Hepatobiliary and Pancreatic Surgery, The Affiliated Hospital of Qingdao University, 16 Jiangsu Street, Qingdao, 266005 China; 2 Organ Transplantation Center, The Affiliated Hospital of Qingdao University, Qingdao, Shandong China; 3 Department of Surgery, Asian Liver Center, Stanford University School of Medicine, Stanford, CA USA

**Keywords:** Hepatocellular carcinoma, Immune-related genes (IRGs), Tumor immune microenvironment(TIME), Cancer immunotherapy, Prognostic model

## Abstract

**Background:**

The combined application of immune cells and specific biomarkers related to the tumor immune microenvironment has a better predictive value for the prognosis of HCC. The purpose of this study is to construct a new prognostic model based on immune-related genes that regulate cross-talk between immune and tumor cells to assess the prognosis and explore possible mechanisms.

**Method:**

The immune cell abundance ratio of 424 cases in the TCGA-LIHC database is obtained through the CIBERSORT algorithm. The differential gene analysis and cox regression analysis is used to screen IRGs. In addition, the function of IRGs was preliminarily explored through the co-culture of M2 macrophages and HCC cell lines. The clinical validation, nomogram establishment and performing tumor microenvironment score were validated.

**Results:**

We identified 4 immune cells and 9 hub genes related to the prognosis. Further, we identified S100A9, CD79B, TNFRSF11B as an IRGs signature, which is verified in the ICGC and GSE76427 database. Importantly, IRGs signature is closely related to the prognosis, tumor microenvironment score, clinical characteristics and immunotherapy, and nomogram combined with clinical characteristics is more conducive to clinical promotion. In addition, after co-culture with M2 macrophages, the migration capacity and cell pseudopod of MHCC97H increased significantly. And CD79B and TNFRSF11B were significantly down-regulated in MHCC97H, Huh7 and LM3, while S100A9 was up-regulated.

**Conclusion:**

We constructed an IRGs signature and discussed possible mechanisms. The nomogram established based on IRGs can accurately predict the prognosis of HCC patients. These findings may provide a suitable therapeutic target for HCC.

**Supplementary Information:**

The online version contains supplementary material available at 10.1186/s12885-022-09465-9.

## Introduction

Hepatocellular carcinoma (HCC) ranks third in the global cancer-related mortality rate [1], usually caused by chronic hepatitis and liver fibrosis [2, 3]. Surgical resection and liver transplantation are often used as the two main treatments for HCC. However, due to the shortage of liver donors and the high recurrence rate of patients, the overall prognosis is not satisfactory [4]. In China, HCC is usually diagnosed at a late stage, which leads to the existing treatment methods with greater limitations and poor results. Less than 14.1% of patients live for up to 5 years [5]. Therefore, there is a need for early prediction of the survival status of patients, exploring new treatment methods to provide patients with personalized treatment and improving the clinical prognosis of patients.

Some studies have shown that immunotherapy has shown broad application prospects in treating many advanced cancers, especially for virus-induced cancers [6]. In China, most HCC patients are associated with HBV and suffer from chronic hepatitis. Meanwhile, the liver is considered to be an immune-tolerant organ. It can limit hypersensitivity to antigens and bacteria through the portal vein and can effectively receive allogeneic liver transplantation, creating an immunosuppressive microenvironment for the liver [7]. This shows that HCC patients may be more appropriate for immunotherapy.

The development of immunotherapy focuses on the tumor immune microenvironment (TIME) [8]. In addition to tumor cells, various immune cells, mesenchymal cells, secreted cytokines, chemokines and other non-tumor components that are also infiltrated in TIME have shaped different tumor heterogeneities [9, 10]. It has been reported that the HCC TIME has varieties of cytokines and is closely relevant to the prognosis of patients in many research, such as IL-6, IL-10, etc. [11, 12]. Therefore, we believe that the different cytokines and cell components in TIME have important guiding significance for the prognosis of patients. However, there is still a lack of immune-related genes that regulate the immune and tumor cells to effectively assess the heterogeneity of TIME and the prognosis of patients. Therefore, looking for key immune genes as HCC markers, clinicians can better understand the immunological characteristics of HCC and provide directions for patient prognosis and immunotherapy [13].

In this study, we downloaded the clinical survival information and RNA expression data of 424 cases in the Tumor Genome Atlas (TCGA-LIHC) database, and analyzed the content of 22 immune cells in the patients based on the CIBERSORT algorithm. Four immune cells related to survival in HCC were identified. And we also screened three IRGs that regulate the level of immune cell immersion. In the validation study, we chose M2 macrophages and three HCC cell lines as our cell models. We reported three essential IRGs that regulate the "cross-talk" between immune cells and tumor cells in TIME. Subsequently, we constructed a prognostic nomogram combining IRGs signature and clinical factors, which guides forecasting the prognosis of patients, and it may be a proper therapeutic target for HCC patients.

## Materials and methods

### Data source

From the Cancer Genome Atlas (TCGA) data portal, we downloaded the RNA-Seq gene expression profiles (FPKM and COUNT format) of 374 HCC and 50 adjacent normal HCC tissues, as well as clinical data on patient age, survival time, tumor staging, etc. In addition, RNA-Seq gene expression profiles and clinical information on 243 HCC specimens were validated from the ICGC database and 115 HCC specimens from the GEO database (GSE76427), respectively.

### Identifying survival-related immune cells

The RNA-Seq (FPKM format) of 424 specimens were analyzed using the CIBERSORT algorithm and obtained a ratio matrix of 22 immune cells (perm = 100) [14, 15]. Owing to those samples with CIBERSORT *P*-value > 0.05 may represent samples with low immune cell infiltrate, they cannot be ignored. Therefore, we select 127 samples with CIBERSORT *P*-value < 0.1 and follow-up days ≥ 30 days for follow-up analysis [16]. Then, we analyzed the correlation in 22 immune cells in 127 patients. Finally, the Kaplan–Meier analysis for overall survival was used to identify survival-related immune cells, whose cut-off level was set at the median value according to the abundance ratio of 22 immune cells. Through using independent sample t-test and one-way ANOVA test, we analyzed the relationship between the abundance ratio of immune cells and tumor grade, clinical stage, and stage T.

### Identifying Differentially expressed Immune-Related Genes (DEIRGs)

Cox proportional hazards regression was established based on the four survival-related immune cells identified in the previous steps. The 127 samples were sorted into high-risk (*n* = 64) and low-risk (*n* = 63) groups based on risk scores. We got 2498 unique immune-related genes from Immport database (https://www.immport.org/home), and we established the expression matrix of immune-related genes in 127 samples (count format) [17]. Through the edgeR R package for analysis of DEIRGs with the conditions: |logFC|> 1 and *P* < 0.05 [18].

### Protein–Protein Interaction Network Construction and Hub Genes Screening

The 412 differential IRGs were analyzed in the STRING database (https://stringdb.org/), with the condition: combined-score ≥ 0.4 [19]. This network was visualized using Cytoscape 3.8.2 and analyzed by the MCODE plugin. Ultimately, we obtained 11 modules and 10 seed genes. At the same time, Cytohubba was used to screen the top 20 nodes ranked by degree. We selected 30 genes as immune microenvironment-related hub genes.

### Relationship between clinical characteristics and hub genes

For our study, 127 patients were grouped and using Kaplan–Meier survival analysis, and the overall survival rate was analyzed according to the expression level of the 30 hub genes. Here, we identified 9 survival-related hub genes. We analyzed and visualized the hub genes’ connection with clinical characteristics by the "WGCNA" R package.

### Construction of the IRGs signatures

To develop a prognostic model, 9 survival-related genes in Kaplan–Meier survival analysis were included in multivariate proportional hazards regression analysis. The 127 patients were sorted according to their risk score, which was derived from gene expression multiplied by a linear combination of regression coefficients obtained from the multivariate Cox regression. The 63 patients with the low-risk score were defined as the low-risk group, and the remaining 64 patients were in the high-risk group. Using the Kaplan–Meier analysis to compare OS between the two groups of patients and the "survival ROC" package to plot receiver operating characteristic (ROC) curve.

### External validation of the IRGs

243 HCC specimens in the ICGC database and 115 HCC specimens in the GSE76427 were used as a verification cohort to verify the prognostic accuracy of the IRGs signature risk score model. The samples were divided into high-risk and low-risk groups by calculating risk scores based on the same formula, and their Kaplan–Meier and ROC curve were analyzed, respectively.

### Enrichment analysis of differentially expressed genes (DEGs) between low-risk and high-risk groups

Through the edgeR R package for analysis of DEGs between low risk(*n* = 63) and high risk(*n* = 64) groups in TCGA with the conditions: |logFC|> 1 and *P* < 0.05. Analyzing DEIRGs in the GO (Gene Ontology) and KEGG (Kyoto Encyclopedia of Genes and Genomes) pathways via the DAVID 6.8 (https://david.ncifcrf.gov/) [20, 21]. The GO terms and KEGG signaling pathways are then visualized via R Package "ggplot2" with the conditions: FDR < 0.05 and counts ≥ 4.

### Analysis of the degree of immune infiltration between low-risk and high-risk groups

The ssGSEA was executed to probe into the different infiltration degrees of immune cell types, immune-related functions and pathways in the expression profile of low-risk and high-risk groups using the R package "GSVA" based 29 immune-related gene sets [22]. To prove the effectiveness of IRGs risk scores and to picture clustering heatmap, we made use of R package "ESTIMATE" to study the expression level of RNA-seq to count the tumor purity, estimate score(ES), immune score(IS), and stromal score(SS). Using the R package "ggpubr", we obtained the vioplots of ES, IS, and SS in low-risk and high-risk groups. The correlation of immune cells with IRGs signature risk score was analyzed and visualized by the "corrplot" package in R.

### Construction of prognostic nomogram

To provide a quantitative analysis tool to predict the survival risk of HCC patients, we were further constructed the nomogram on the basis of IRGs as well as clinical parameters in 127 patients in TCGA. In order to evaluate the accuracy of the nomogram, we used 115 patients in GSE76427 for external validation, and the calibration curve and DCA curves are drawn with the R-pack "rms" and "ggDCA".

### Cell line culture

MHCC-97H, Huh7, LM3 HCC cell lines and THP-1 cell line were purchased from the Shanghai cell bank (Chinese Academy of Sciences, Shanghai, China). HCC cell lines were cultured in a medium with 10% FBS and 1% P/S (Gibco, Thermo Fisher Scientific, Waltham, USA), and THP-1 cells were cultured in RPMI 1640 medium (Hyclone, Thermo Fisher Scientific, Waltham, USA) with 10% FBS. All cells were maintained in a humidified atmosphere with 5% CO2 at 37 °C.

### THP-1-derived M2 macrophages and Establishment of co-culture system

THP-1 cells were treated with phorbol 12-myristate 13-acetate (PMA) (Sigma, Saint-Quentin Fallavier, France, 100 ng/mL) for 24 h to polarize THP-1 cells into macrophages. The IL-4 and IL-13 were then polarized into M2 macrophages (Sino Biological Als, China, 5 μg). MHCC-97H cells (1 × 10^6^ cells) were placed in the lower chamber of a 6-well transwell plate. After 24 h, M2 macrophages (1 × 10^6^ cells) derived from THP-1 were placed on the 0.4-μm porous membrane in the upper chamber to establish a co-culture system in a serum-free DMEM medium [23]. Then 48 h later, MHCC-97H cells were collected for RNA extraction and other experiments.

### Cell migration assay

Using transwell compartments (8 um pore) to assess the cell migration capacity (Corning, 353,097). Normal MHCC-97H cells and M2 macrophages co-culture treated MHCC-97H cells (5 × 10^4^ cells) were suspended in serum-free medium in the upper compartment of a 24-well transwell plate, while medium with 30% FBS is placed in the lower chamber. After 24 h at 37 °C, the translocated cells were stained with 0.5% crystal violet for 20 min.

### Quantitative real-time PCR (qPCR)

Total RNA was extracted with RNA-easy isolation reagent (Vazyme, Nanjing, China). PrimeScript™ RT Kit (TaKaRa, RR047A) and SYBR Premix EX Taq™ (TaKaRa, RR820A) were used to cDNA synthesis and qPCR on the FTC-3000P real-time PCR system (Funglyn Biotech, Shanghai, China). Supplementary Table 1 shows the PCR primers used.

### Statistical analysis

Using IBM SPSS Statistics performed all analyses (version 23). A *P* < 0.05 indicated statistical significance.

## Results

### Identifying survival-related immune cells

**Fig. 1:**
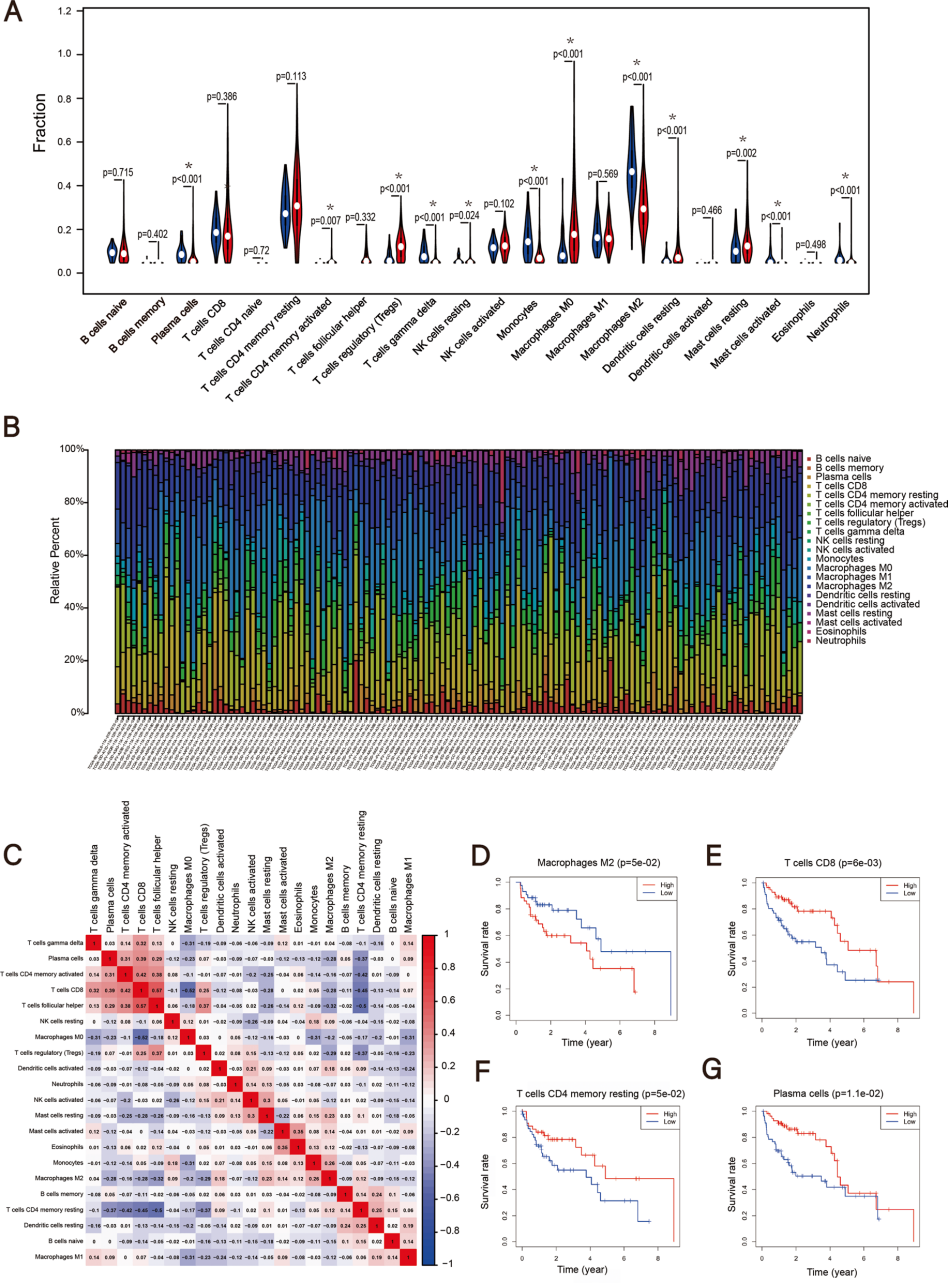
The relationship between the abundance ratios of immune cells and overall survival. **A** Differences in the expression of 22 immune cells in adjacent normal HCC and HCC tissue. **B** The abundance ratio of immune cells in the 127 samples. **C** The relationship between the abundance ratios of various immune cells. **D**–**G** The survival analysis for the abundance ratios of the four immune cells

Using the CIBERSORT algorithm to analyze the abundance ratio of 22 immune cells in 127 samples, revealing the relative content of 22 immune cells in normal and tumor samples (Fig. 1A, 1B). As shown in Fig. 1A, M0/M1/M2 Macrophages, CD8 + T cells, and dendritic cells occupy a large proportion in the sample. In adjacent normal HCC tissues, the content of active mast cells, M0 macrophages and Tregs were significantly higher than that of tumor samples (*P* < 0.05), while the content of M2 Macrophages, plasma cells and monocytes were significantly lower than that of tumor samples (*P* < 0.05) (Fig. 1B). Correlation analysis further suggests that there are connections between 22 immune cells (Fig. 1C). CD8 + T cells are positively correlated with the content of T cells follicular helper, active CD4 + T memory cells, and Plasma cells, but negatively correlated with the content of resting CD4 + T memory cells, M0 macrophages, and M2 macrophages. In addition, Fig. 1D-G show that the abundance ratios of the four types of immune cells are related to survival rates by Kaplan–Meier analysis, among CD8 + T cells (*P* = 0.006), Plasma cells (*P* = 0.01), and CD4 + memory resting T cells (*P* = 0.05) are indicators of favorable prognosis, while M2 Macrophages (*P* = 0.05) are indicators of unfavorable prognosis. The correlation between abundance ratios of the four immune cells and clinical characteristics reveals that CD8 + T cells, Plasma cells, and resting CD4 + T memory cells decreased with the increase of stage T, clinical stage, and tumor grade, while M2 Macrophages is the opposite (Supplementary Fig. 1).

### Identifying immune-related genes and enrichment analysis

Cox proportional hazards regression was established based on the four survival-related immune cells. Risk scores = Plasma cells*( -7.76) + CD8 + T cells *( -3.26) + resting CD4 + T memory cells *( -4.42) + M2 Macrophages * 1.08. According to the risk score, the samples were divided into high-risk and low-risk groups. We analyzed the immune-related genes related to the risk score level and obtained 412 immune-related differential genes (Supplementary Fig. 2A). Using the DAVID website, GO/KEGG enrichment performed the analysis of 412 immune-related differential genes. The supplementary Fig. 2B-E shows the top 12 enrichment results. The results showed that the differential genes were mainly located in T cell receptor complex and extracellular exosome, significantly involved in complement activation, inflammatory response, antigen binding, transmembrane signaling receptor activity, and were mainly enriched in the chemokine signaling pathway, natural killer cell-mediated cytotoxicity, Jak-STAT signaling pathway. In conclusion, 412 immune-related gene proteins are mainly involved in various signaling pathways and the regulation of immune responses, cell proliferation and apoptosis, closely connecting various immune cells, stromal cells and tumor cells in the tumor microenvironment.

### Protein–protein interaction network construction and hub genes screening

**Fig. 2:**
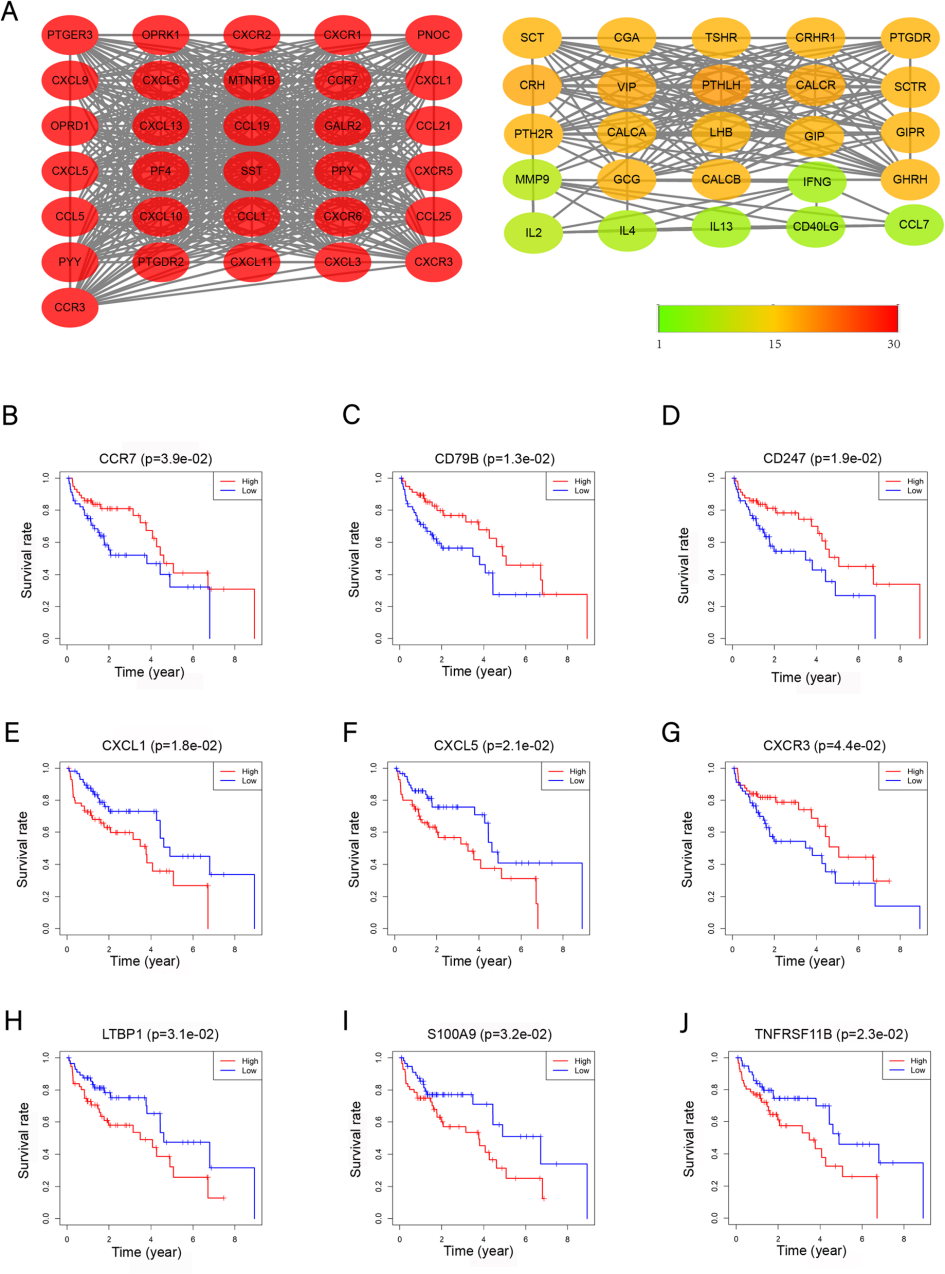
The top two modules and survival analyses of the hub genes. **A** Two modules in MCODE. Redder indicates that the higher the number of interactions with other proteins, the smaller the greener the number. **B**-**J** The nine genes are significantly related to survival. Red represents high gene expression, and blue represents low gene expression

To probe into the interrelationship between immune-related genes and get hub genes, we performed PPI and module analysis to obtain 30 hub genes. Supplement Table 2 shows the full names and primary functions of 30 hub genes, meanwhile Cytoscape analysis was used to get the first two most important modules (Fig. 2A-B). The functional analysis of genes involved in this module was analyzed using DAVID. Module 1 is mainly related to HIV and lung cancer. It is primarily concentrated in immune cell activation and chemotaxis, and cell proliferation (Table 1). Module 2 is mainly related to HCC, HBV infection, lung cancer and is primarily enriched in the proliferation and differentiation of immune cells and apoptosis (Table 1). Both are closely related to cancer and immune signaling pathways, such as the chemokine signaling pathway, Jak-STAT signaling pathway, TNF signaling pathway.

**Table 1:** GO and KEGG pathway enrichment analysis of the top 2 modules

Modules	Description	*P*.adjust	Count
Module 1			
BP terms	positive regulation of ERK1 and ERK2 cascade	1.34E-05	6
	chemotaxis	2.89E-27	17
	regulation of cell proliferation	7.86E-07	7
	positive regulation of JNK cascade	0.00598	3
KEGG pathway	Chemokine signaling pathway	3.09E-27	21
	TNF signaling pathway	0.00733	4
Module 2			
BP terms	positive regulation of cell proliferation	2.78E-06	8
	G-protein coupled receptor signaling pathway	1.91E-04	8
	negative regulation of apoptotic process	0.00367	5
KEGG pathway	T cell receptor signaling pathway	0.00207	4
	Jak-STAT signaling pathway	0.00593	4
MF terms	hormone activity	4.66E-13	9

### Relationship between clinical characteristics and hub genes

Through Kaplan–Meier survival, we analyzed 30 hub genes, and obtained 9 immune-related genes with prognostic significance (*P* < 0.05), Including CCL5, CCR7, CD79B, CD247, CXCL1, CXCL5, CXCR3, LTBP1, S100A9, TNFRSF11B (Fig. 2B-J). Table 2 shows the correlation analysis between these nine prognostic-related hub genes and their clinical characteristics. CCR7 and CD79B have a significant positive correlation with stage (I/II/III/IV) and stage T, and LTBP1 has a significant negative correlation with stage.

**Table 2:** The correlation between the 24 hub genes and clinical characteristics

Gene **Cor(*P*-value)**	Age	Gender	Grade	Stage(I/II/III/IV)	T stage
CCR7	0.994(0.323)	0.789(0.435)	1.741(0.086)	2.263(0.026)	2.223(0.029)
CXCL1	0.52(0.604)	-0.359(0.720)	-1.719(0.093)	-1.153(0.257)	-1.13(0.267)
CXCR3	0.952(0.343)	0.352(0.726)	-0.322(0.749)	0.476(0.636)	0.408(0.685)
CXCL5	0.92(0.360)	-0.986(0.326)	-1.501(0.141)	-1.339(0.190)	-1.351(0.187)
TNFRSF11B	0.172(0.864)	-0.79(0.431)	-0.507(0.614)	-0.297(0.769)	-0.341(0.735)
CD247	0.634(0.527)	1.032(0.308)	-0.099(0.921)	0.808(0.422)	0.713(0.478)
S100A9	0.588(0.558)	-0.251(0.803)	-0.194(0.846)	0.393(0.695)	0.338(0.736)
LTBP1	-0.675(0.502)	1.013(0.316)	-0.917(0.363)	-2.286(0.029)	-1.666(0.104)
CD79B	1.312(0.193)	0.422(0.675)	0.53(0.597)	2.251(0.027)	2.197(0.031)

### Establishment and verification of IRGs signature

9 hub genes were tested for their prognostic significance to perform univariate COX analysis and included hub genes with *P* < 0.1 into the multivariate COX analysis (Table 3). To get the best model, these 9 genes were analyzed using the Cox proportional hazards model method of R package "survival". Finally, 3 immune-related genes were used to construct Cox proportional hazards model as follows: Riskscore = CD79B*(-0.00158) + TNFRSF11B*0.0000946 + S100A9*0.000025.

**Table 3:** Univariate and multivariate analysis of 9 hub genes with OS

Gene	Univariate analysis		Multivariate analysis	
	*P* value	Hazard ratio	*P* value	Hazard ratio
CD79B	**0.025**	0.804(0.664–0.974)	0.030*	0.803(0.659–0.979)
CXCL1	0.675	0.998(0.992–1.005)		
CD247	**0.059**	0.754(0.562–1.011)	0.738	0.938(0.654–1.363)
CXCL5	0.259	1.005(0.996–1.012)		
TNFRSF11B	**0.018**	1.011(0.994–1.028)	0.027*	1.008(0.991–1.025)
LTBP1	0.427	1.020(0.971–1.071)		
S100A9	**0.006**	1.000(1.000–1.000)	0.003*	1.001(0.999–1.001)
CCR7	**0.057**	0.793(0.624–1.007)	0.814	0.959(0.679–1.355)
CXCR3	0.220	0.917(0.799–1.053)		

**Fig. 3:**
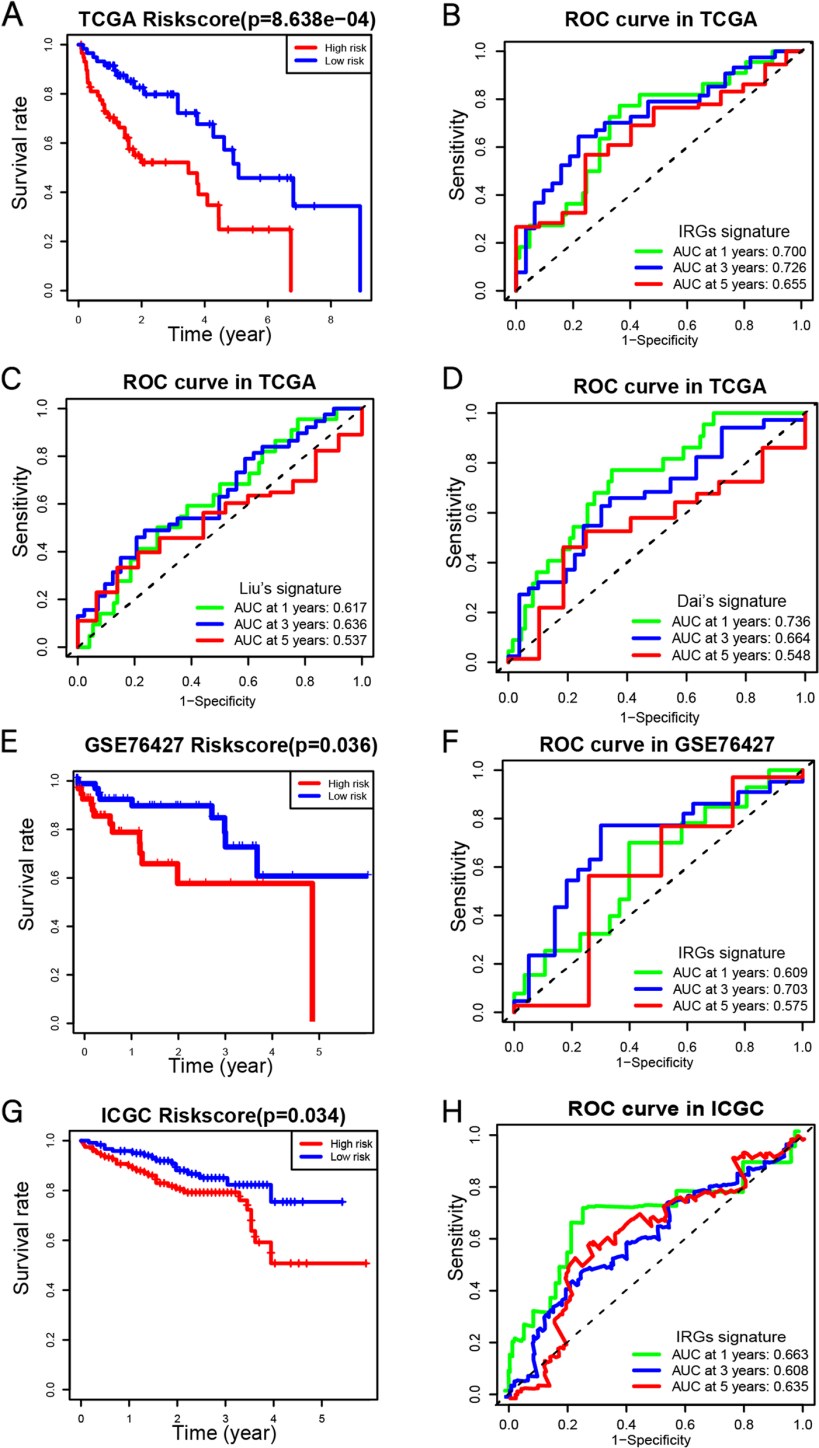
IRGs signature accurately predicts survival of HCC patients. The Kaplan–Meier curve of the overall survival between the high-risk and low-risk groups was stratified by the median risk score in TCGA (**A**), GSE76427 (**E**) and ICGC (**G**). 1-year, 3-year, 5-year ROC analysis of the predictive efficiency of the IRGs signature (**B**), Liu’s signature (**C**) and Dai’s signature (**D**) on overall survival based on risk score in TCGA. 1-year, 3-year, 5-year ROC analysis of the predictive efficiency of the IRGs signature on overall survival based on risk score in GSE76427 (**F**) and ICGC (**H**)

Further, the risk scores of 127 HCC patients are sorted and divided into high-risk(*n* = 64) and low-risk(*n* = 63) groups according to the median risk score. Through K-M analysis, the higher the risk score of patients, the worse the prognosis (*P* < 0.001, Fig. 3A). We plotted not only the ROC curves of our IRGs signature, but also the ROC curves of other previously published IRGs signatures (Liu’s signature [13] and Dai’s signature [24]), which have been published previously (Fig. 3B-D). Interestingly, the 1-year, 3-year and 5-year AUC of our IRGs signature was significantly higher than Liu’s signature and Dai’s signature in the training cohort. These prove the excellent value of the IRGs we constructed in predicting the prognosis of HCC patients.

### External validation of the IRGs signature

External verification was performed on 243 HCC patients in the ICGC database and 115 HCC patients in the GSE76427. Sorted based on risk scores, patients were divided into high-risk (*n* = 121 in ICGC, *n* = 57 in GSE76427) and low-risk groups (*n* = 122 in ICGC, *n* = 58 in GSE76427) with the median risk score as the cut-off value. K-M analysis showed that patients in high-risk groups had a worse prognosis. (Fig. 3E, G). The ROC curve analysis was performed, and the results showed that the AUC values ​​of 1, 3, and 5 years were 0.663, 0.608, and 0.635 respectively, and 0.609, 0.703 and 0.575 in the GSE76427 respectively (Fig. 3F, H). This proves that the IRGs signature has a strong predictive ability.

### Identifying DEGs between high-risk and low-risk groups and Enrichment Analysis

**Fig. 4:**
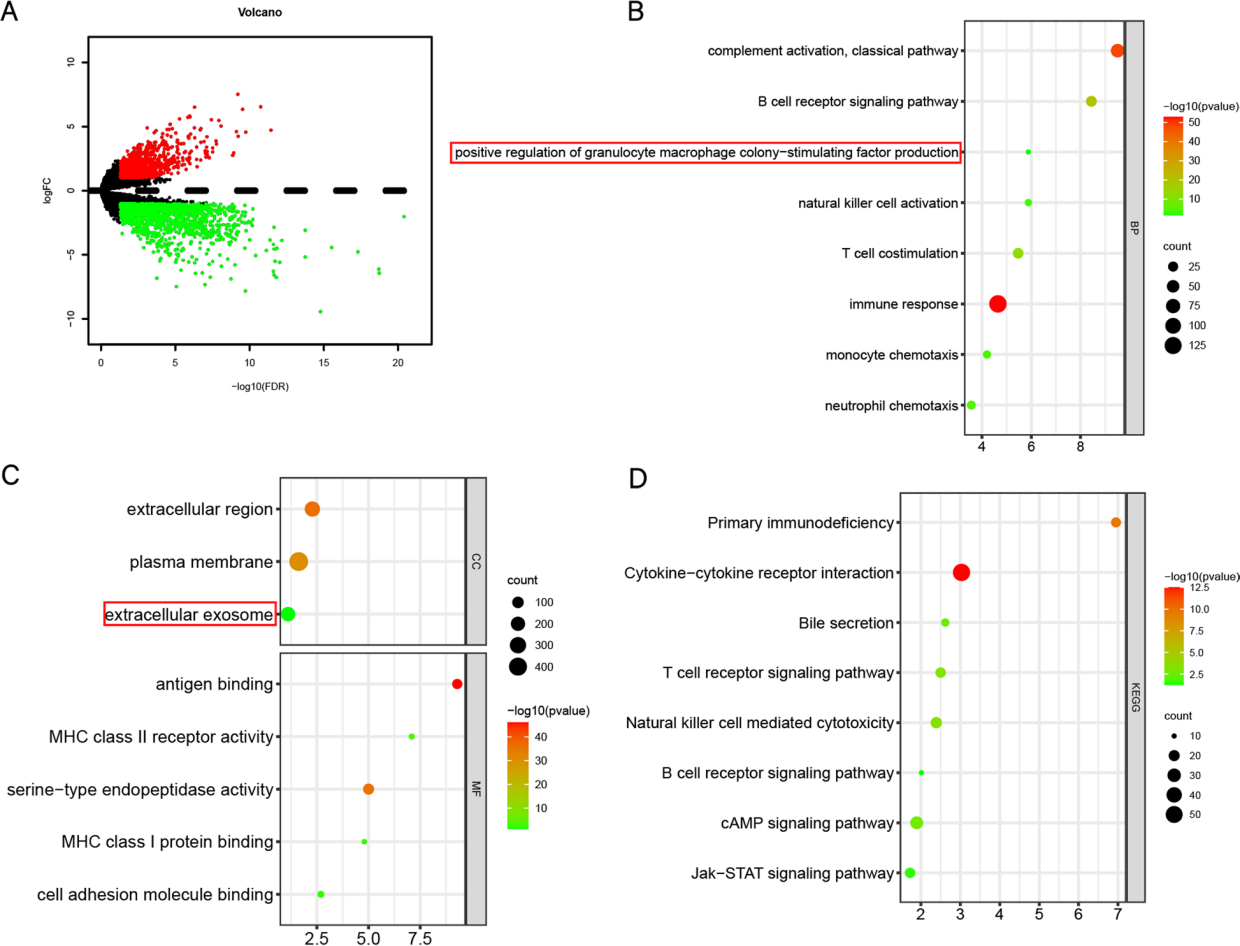
Enrichment analysis of DEGs. **A** The volcano shows DEGs between low-risk and high-risk groups. **B**-**D** Represent the enrichment analysis results of DEGs involved in IRGs signature, namely biological processes, cellular components, molecular functions, and KEGG

Through differential expression analysis between low-risk and high-risk groups, we obtained 2371 DEGs (Fig. 4A). Interestingly, GO analysis results showed that the changes in the biological process (Fig. 4B) were significantly enriched in immune response, particularly in the B cell receptor signaling pathway, T cell co-stimulation and granulocyte–macrophage colony-stimulating factor production. The cellular component which has changes (Fig. 4C) was mainly enriched in the extracellular region, plasma membrane, extracellular exosome. KEGG pathway (Fig. 4D) was mainly enriched in natural killer cell-mediated cytotoxicity, T cell receptor signaling pathway, Jak-STAT signaling pathway. These results suggest that our IRGs signature regulates the immune response primarily by participating in stimulation or activation of T cell and B cell, as well as the polarization of macrophages.

### The degree of immune infiltration between low-risk and high-risk groups

**Fig. 5:**
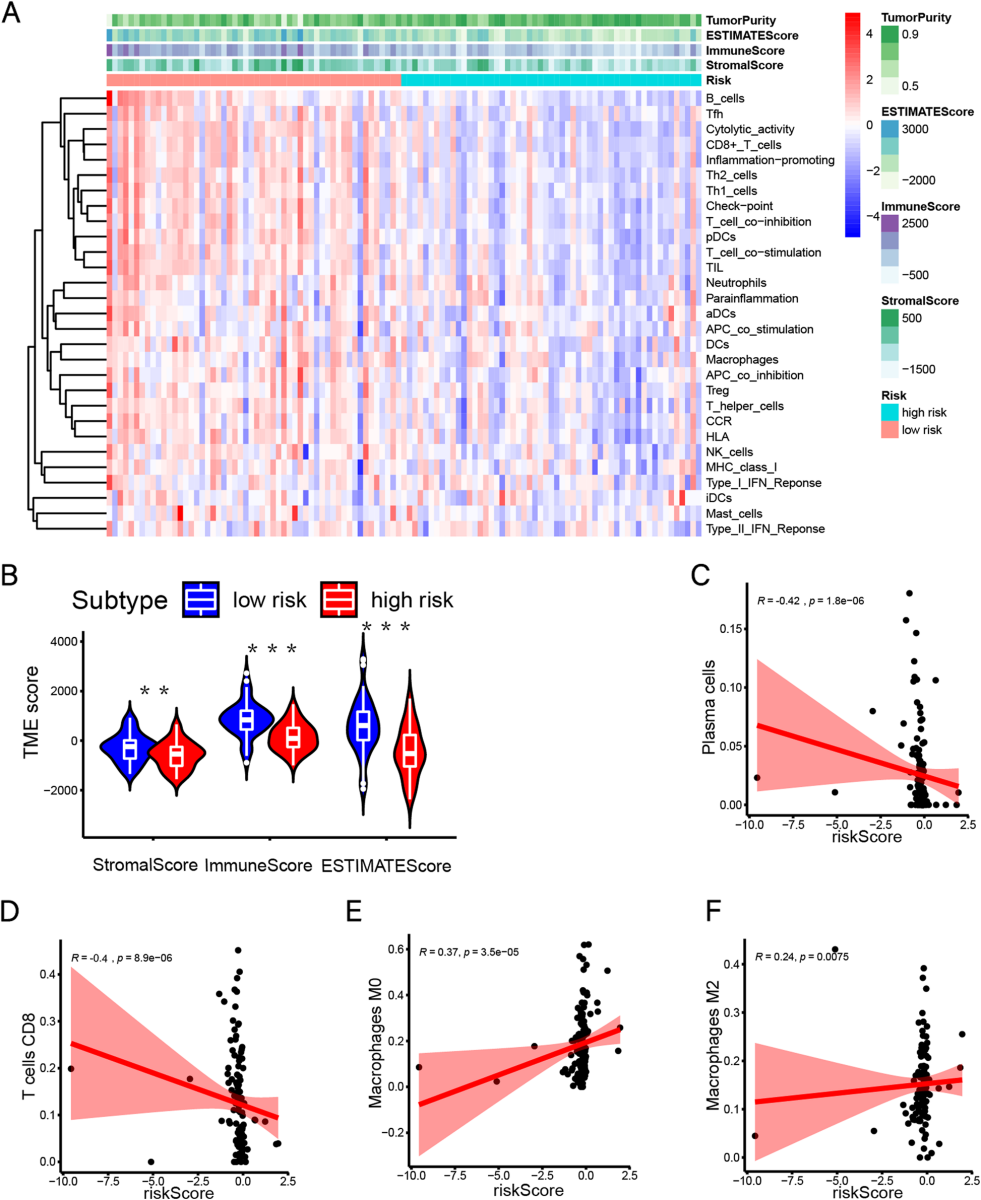
The degree of immune infiltration between low-risk and high-risk groups. **A** Differences in 29 immune-related gene sets between the high-risk and low-risk groups. Using ESTIMATE algorithm to calculate the SS, IS, ES. **B** The violin plots of SS, IS, ES. **C**-**F** Spearman’s correlation analysis of immune cells and IRGs signature risk scores

The RNA sequencing data of HCC samples were analyzed by the ssGSEA method and got the abundance levels of 29 immune-related cells and types in 127 HCC samples. Patients in the low-risk group had a higher degree of immune cell infiltration (Fig. 5A). Meanwhile, we calculate the stromal score (SS), immune score (IS) and estimate score (ES) by using the ESTIMATE algorithm. The result has shown that the SS, IS, and ES of the low-risk group was significantly higher than that of the high-risk group (*P* < 0.05) (Fig. 5A-B). Immune cell correlation analysis showed that as the risk score increased, plasma cells and CD8 + T cells gradually decreased, and conversely M2 cells and M0 cells gradually increased (*p* < 0.05) (Fig. 5C-F).

### Establishment of a risk-nomogram for predicting survival in HCC patients

**Fig. 6:**
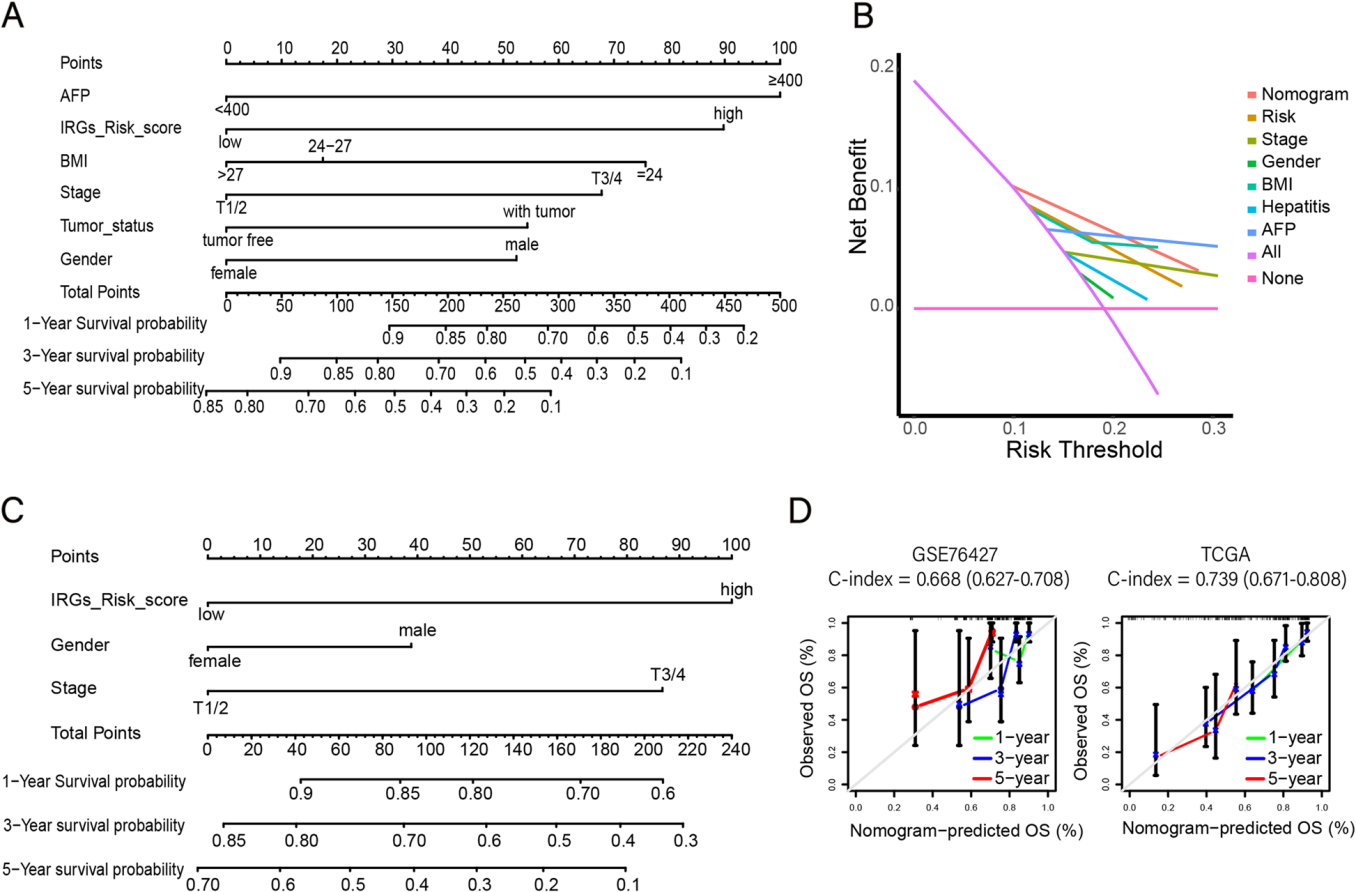
Construction of nomograms in the training cohort and the validation cohort. Construction of nomograms in the training cohort of TCGA (**A**) and the validation cohort of GSE76427 (**C**). **B** DCA curves show the clinical utility of the nomogram models other models for the 3-year OS. (**D**) The calibration plots for predicting the 1-year, 3-year and 5-year OS rates, with an accompanying C-statistic discriminatory index value

Combined with other univariate and multivariate COX analyses of significant and important clinical features (*P* < 0.1), such as AFP level, hepatitis, tumor status and other factors that help in the disease detection, we have established a convenient and clinically adaptable risk nomogram to predict the survival probability of HCC patients in the training cohort of TCGA and the validation cohort of GSE76427. By calculating the sum of the scores corresponding to the corresponding levels of each factor in the nomogram, the higher the total score, the worse the patient’s 1-year, 3-year, and 5-year OS rate (Fig. 6A, C).

To verify the validity of this nomogram, we calculated the C-statistic discriminatory index and the calibration plot of the prediction models in the training cohort and the validation cohort. Figure 6D shows the calibration plot for predicting the 1-year, 3-year and 5-year OS rates, with an accompanying C-statistic discriminatory index value of 0.739 and 0.668, demonstrating the good predictive ability and effectiveness of our nomograms. This proves that the IRGs signature combines clinical characteristics such as AFP levels and hepatitis to further enhance clinical value and predictive power (Table 4). In addition, according to the DCA curve, the nomogram showed exceptional performance in the training cohort (Fig. 6B).

**Table 4:** Univariate and multivariate analysis of IRGs signature and clinical characteristics with OS

Gene	Univariate analysis		Multivariate analysis	
	*P* value	Hazard ratio	*P* value	Hazard ratio
BMI	0.037	0.693(0.491–0.979)	0.095	0.718(0.412–1.062)
Age	0.941	0.985(0.670–1.450)		
Gender	0.524	1.228(0.652–2.312)	0.088	1.790(0.922–3.518)
Grade	0.933	1.013(0.754–1.360)		
Stage	0.014	1.452(1.080–1.952)		
Stage T	0.008	1.493(1.110–2.008)	0.064	1.36(0.98–1.89)
IRGs risk score	0.002	2.602(1.437–4.709)	0.022	2.15(1.12–4.13)
Tumor status	0.081	1.652(0.939–2.906)	0.017	2.48(1.18–5.24)
Family cancer history	0.671	1.130(0.642–1.998)		
AFP	< 0.001	2.619(1.489–4.607)	0.012	2.24(1.20–4.21)
Hepatitis B/C	0.088	0.614(0.351–1.075)	0.035	0.73(0.37–1.42)

### Effects of M2 macrophage levels on HCC cell migration

**Fig. 7:**
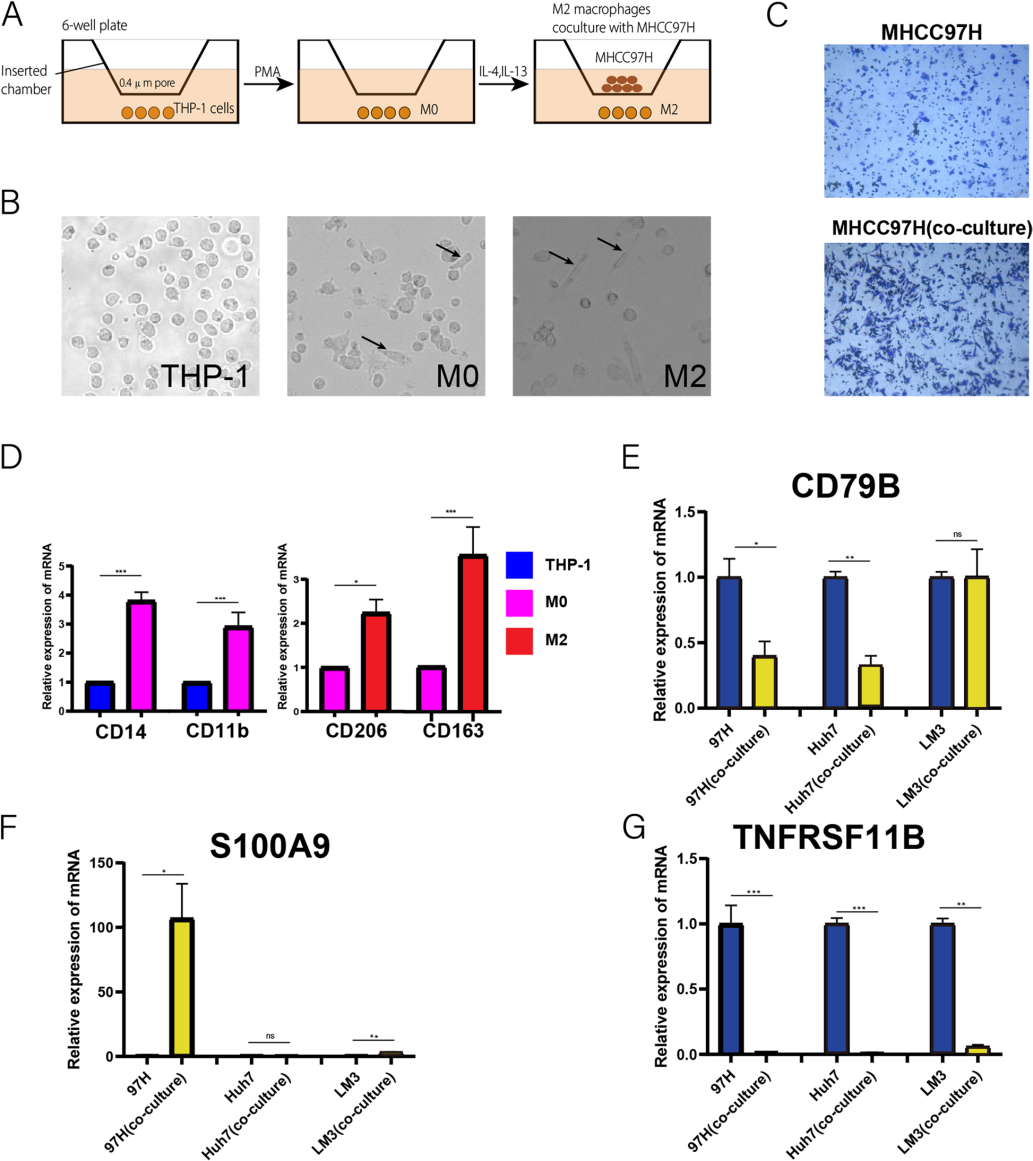
Polarization of THP1 cells and co-culture experiment. **A** Schematic model co-culture system. **B** The morphological differences in THP1, M0, and M2 cells under a light field. **C** Comparison of the MHCC-97H migration using transwell compartments. **D** Detect M0 and M2 macrophage markers by qPCR to determine the polarization state of macrophages. **E**–**G** The expression of IRGs signature genes in the co-culture group and three normal HCC cell lines were detected by qPCR. * *P* < 0.05, **P < 0.01, *** *P* < 0.001

We used PMA, IL-4 and IL-13 to induce M2 macrophage formation (Fig. 7A). First, the M0 macrophage markers CD11b and CD14 were detected by qPCR. Then, the expression of M2 macrophage marker CD206 and CD163 was significantly up-regulated compared to M0 macrophages by qPCR (*P* < 0.05) (Fig. 7D). Figure 7B shows morphological changes in THP-1, M0 and M2 cells. We then probe into whether M2 macrophages affected HCC cell migration levels. After co-culture with M2 macrophages, we observed a significant increase in the ability of MHCC-97H to migrate, and the pseudopod of MHCC-97H cells has increased significantly in morphology (Fig. 7C). It is proved that M2 macrophages increase the malignancy of HCC.

### IRGs identified as key factors of tumor microenvironment in HCC

To determine the essential value of CD79B, S100A9, TNARSF11B in the immune infiltration of HCC. Compared with three normal HCC cell lines, in HCC cell lines co-cultured with M2 macrophages, the expression of CD79B and TNFRSF11B was significantly down-regulated, while the expression of S100A9 was up-regulated considerably (Fig. 7E-G).

## Discussion

The liver receives blood from the hepatic artery and portal vein and has a rich blood supply [25]. Therefore, the liver will be exposed to intestinal-derived microorganisms and food-derived harmless antigens at a high level for a long time, giving the liver a unique immune privilege [7]. Despite a major histocompatibility complex (MHC) mismatch, the liver can accept allogeneic transplantation by inducing immune tolerance [26, 27]. However, the survival rate and activity of hepatitis viruses, bacteria and tumors in the immunosuppressive microenvironment are higher, leading to persistent infections and rapid cancer progression. Various components of the immune microenvironment regulate the occurrence and progress of HCC [28–30]. In recent years, immunotherapy has made great breakthroughs in tumor treatment, including HCC [31, 32]. Considering the close connection between the immune microenvironment in tumorigenesis and immunotherapy [33]. This article hopes to provide guidance for the prediction of prognosis of HCC patients and probe into potential immunotherapy targets for HCC by screening immune cell and gene targets closely related to immune infiltration and clinical characteristics.

**Fig. 8:**
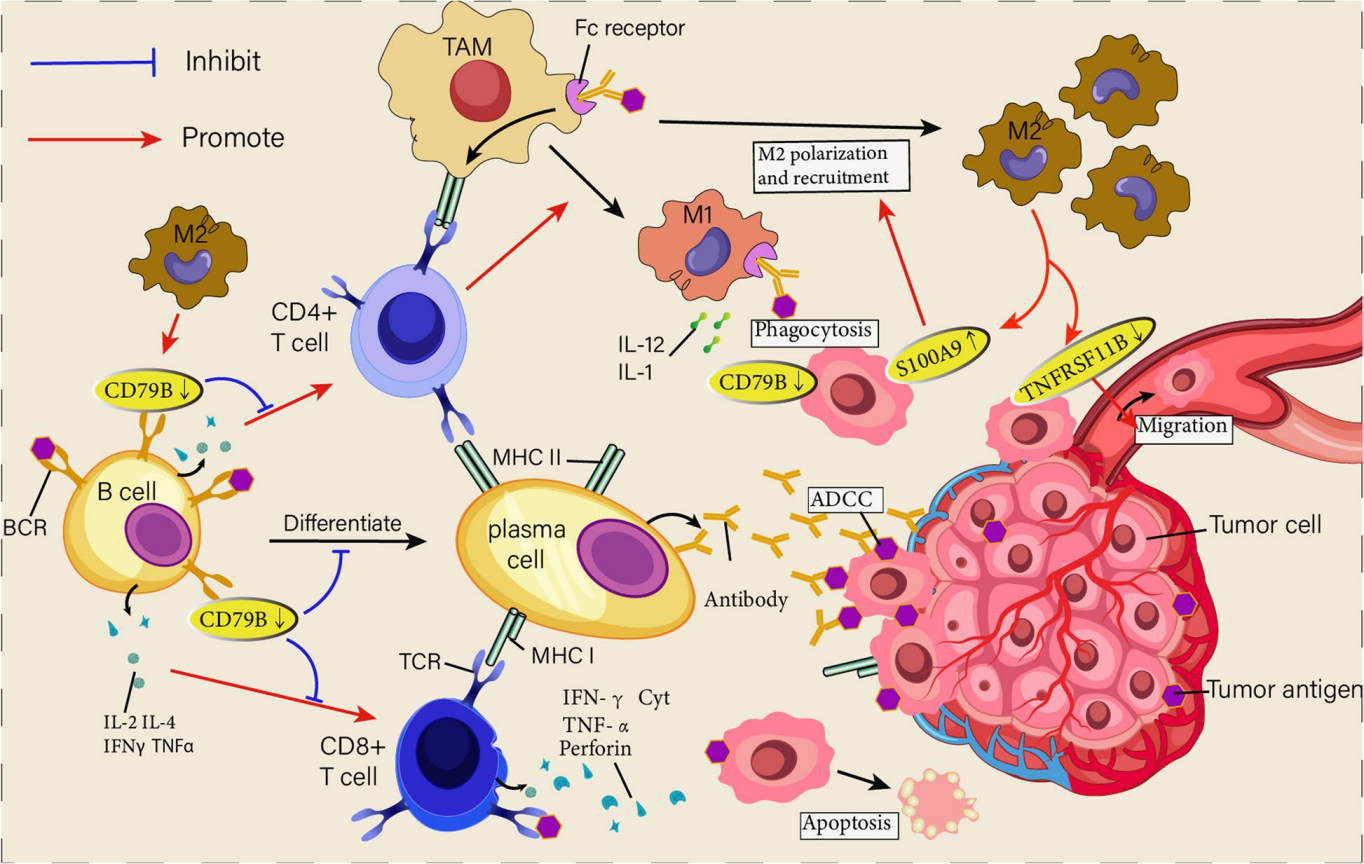
schematic overview. Schematic overview of functional interactions between B cells, PC, T cells and macrophages in the tumor microenvironment. B cells can enhance T cell responses by producing IL-2, IL-4 and other cytokines. Meanwhile, it can be differentiated into PC, which produces antibodies against tumor-associated antigens and triggers antibody-dependent cellular cytotoxicity (ADCC) responses. It also supports tumor-associated macrophages (TAMs) to take up tumor antigens and polarize M1 type to exert phagocytosis. Tumor induces the formation of M2 macrophages, which can increase the expression of tumor cells S100A9, further enhance M2 polarization and recruit M2 macrophages. It can also reduce the expression of TNFRSF11B and enhance the migration ability of tumors. Meanwhile, it can reduce the expression of CD79B, a component of the BCR complex, and weaken the immune function of B cells

The interaction of various components in TIME leads to complex functions of the body. Consequently, understanding the relationship between immune infiltration and patient prognosis is an urgent goal. This study first explored the relationship between M2 macrophages, plasma cells, resting CD4 + T memory cells, CD8 + T cells immune cells and the survival of HCC patients. CD4 + T cells and CD8 + T cells can activate the immune system to kill tumor cells, a research hotspot in tumor immunotherapy [34, 35]. CD8 + T cells can differentiate into effector cytotoxic T lymphocytes (CTLs), which have two main ways to kill tumor cells: granular exocytosis and Fas ligand (FasL)-mediated apoptosis induction [36] (Fig. 8). Most effector cells can return to a resting state and form memory cells [37]. CD4 + T memory cells are essential for adaptive immunity [38] and can be divided into central memory T cell (TCM) and effector memory T cell (TEM) [39]. When re-infected, TEM can release IFN-γ, IL-4 and other cytokines and chemokine receptors in a short period to quickly perform immune functions. TCM can maintain immune memory while expressing IL-2 and dividing rapidly. In the peripheral blood of patients with advanced cancer, the proportion of TCM cells decreases, showing a state of T cell exhaustion [40]. Our research suggests that patients with low levels of resting CD4 + T memory cells and CD8 + T cells have a shorter overall survival period, consistent with the theory of T cell exhaustion. Following antigen exposure and T cell licensing, B cells can secrete IL-2, IL-4, IFN-γ, TNF-α and other cytokines to enhance T cell toxicity [41]. It usually differentiate into potent antibody-secreting plasma cells, which are essential for humoral immunity [42] (Fig. 8). A large amount of evidence indicates that the high expression of plasma cell-related genes is related to the excellent prognosis of various tumors [43, 44]. Tumor-associated macrophages (TAMs) are macrophages that infiltrate around tumor cells. They are mainly divided into classically activated macrophages (M1) and alternately activated macrophages (M2). The overall appearance of the M2 macrophage phenotype promotes immune evasion of tumor cells [45]. The mutual transformation of M1 and M2 macrophages regulates tumorigenesis [46]. Evidence shows that TAMs play an essential role in the development, invasion, drug resistance, immune escape and angiogenesis of HCC [47]. Consistent with previous studies, our study shows that the enrichment of M2 macrophages in HCC indicates a poor prognosis, and the enrichment of plasma cells is an indicator of a favorable prognosis. Overall, the four survival-related immune cells identified in this study are significant in immune infiltration and immunotherapy of HCC, confirming the significance of related gene analysis based on immune cells.

We have identified 30 hub genes, nine of which are related to patient prognosis. Finally, with univariate and multivariate COX regression, we screened 3 Hub genes as an IRGs signature for HCC patients, including S100A9, CD79B, and TNFRSF11B. Previous studies have linked these three genes to tumor development, but the regulatory effect of the HCC immune microenvironment is still unclear. CD79B, as one of the main components of the B-cell antigen receptor complex (BCR), is mainly responsible for transducing antigen-recognition signals to the inside of the cell, expressed on almost all B cell surfaces [48, 49]. BCR signaling primarily affects key functions such as immune synapse formation, antigen affinity, cell migration, etc., and is critical for the maturation and maintenance of B cells [50]. The downregulation of CD79B in the tumor immune microenvironment may lead to abnormal BCR signaling, affecting B cell activity and leading to immunosuppression. S100A9 is a secretory protein in the inflammatory microenvironment that is significantly up-regulated in TAMs and is mainly expressed in neutrophils and circulating monocytes [51, 52] and is related to poor differentiation of HCC, vascular infiltration, invasion and metastasis [53]. Interestingly, M2 macrophages have been reported to secrete more S100A9, enhancing the stem cell-like properties of HCC cells through the AGE/NF-κB axis signaling pathway [54]. TNFRSF11B is able to bind and inhibit TRAIL (TNF-related apoptosis-inducing ligand) to exert anti-apoptotic effects, suggesting that it may provide a survival advantage for cells [55]. It is also controversial in the study of HCC [56, 57]. Studies have shown that in highly aggressive liver cancer cells, the expression of TNFRSF11B is often lower than that of low aggressive liver cancer cells [58]. The same point of view is that the down-regulation of TNFRSF11B expression can promote HCC bone metastasis in vivo [59]. However, there is also evidence that the high expression of TNFRSF11B is an important reason for the high metastatic potential of HCC cells [60]. Hence, we need to study the mechanisms of the three genes further to help develop new HCC treatment strategies.

We have established a new IRGs signature with S100A9, CD79B, and TNFRSF11B. Compared to other previously published IRGs signatures (Liu’s signature [13] and Dai’s signature [24]), our IRGs signature has higher accuracy, and our signature has been successfully verified in the GSE76427 and ICGC databases (Fig. 3). To explore the regulatory role of our IRGs signature, the 127 patients in TCGA were sorted according to the risk score and divided into high-risk(*n* = 64) and low-risk(*n* = 63) groups. Interestingly, GO analysis results the DEGs were significantly enriched in the B cell receptor signaling pathway and T cell co-stimulation, particularly in granulocyte–macrophage colony-stimulating factor production (Fig. 4). And our IRGs signature risk score and M0/M2 macrophages are significantly positively correlated (Fig. 5). This evidence suggests that our IRGs signature is closely related to macrophages.

In addition, immune cells and stromal cells are the two major non-tumor components in the tumor microenvironment. Identifying the right tumor immune subtypes can accurately guide the clinical treatment and prognosis of tumors. We got the abundance levels of 29 immune-related cells between low-risk and high-risk groups (Fig. 5), and found that patients in the low-risk group had a higher degree of immune infiltration. Using ESTIMATE algorithm, we generated immune scores, stromal scores and purity of tumor cells for 127 HCC patients, which have been used to evaluate the immunological characteristics of HCC [61]. Compared to the low-risk group, the immune scores and stromal scores were lower in high-risk group. This suggests that the proportion of immune cells in the tumor microenvironment and tumor purity have an essential impact on the prognosis of HCC. This implies that our IRGs signature has important guiding implications for immunoclassification in HCC patients. For patients with higher risk scores, different immunotherapies combined with conventional treatments should be given priority, to improve the prognosis. Meanwhile, we combined a few selected clinicopathological characteristics, such as BMI, AFP, tumor status, stage, etc., to establish a predictive nomogram model to evaluate the 1-year, 3-year, and 5-year prognosis of HCC patients to achieve accurate prediction of survival. The calibration plots show that the model has a high prediction accuracy. This indicates that the new scoring system established in this study is of great help to the patients who need additional treatment or enhanced follow-up, and can better promote accurate prevention and personalized health management of patients.

We found a significant positive correlation between IRGs signature risk scores and M0/M2 macrophages (Fig. 5E, F). To further explore the functions of the IRGs signature genes associated with M2 macrophages, we first co-cultured three HCC cell lines with M2 macrophages, respectively. The results showed that the migration capacity of MHCC-97H was significantly enhanced, and the pseudopod of MHCC-97H cells has increased significantly in morphology after co-culture with M2 macrophages (Fig. 7C). This proves that M2 macrophages cause changes in gene expression in HCC cells, leading to an increase in the malignancy of HCC. M2 macrophages may have released some macromolecular substances through exosomes that regulate the expression of IRGs signature genes in the microenvironment, such as miR-200c, miR-203 [62]. Considering that IRGs signature genes play a key regulatory role in co-culture systems, we detected changes in the expression of IRGs signature genes in MHCC97H, Huh7 and LM3 cell lines through qPCR. The results showed that in the tumor microenvironment created by M2 macrophages, the expression of CD79B in tumor cells was down-regulated, which may lead to abnormal BCR signaling. Consistent with previous studies [54], the expression of S100A9 in tumor cells was significantly increased after M2 macrophages were co-cultured, and the expression of TNFRSF11B was significantly reduced, which may increase the malignancy of tumor cells (Fig. 8). This implies that we constructed an IRGs signature that may regulate the "cross-talk" between immune cells and tumor cells.

Overall, we identified 4 immune cells related to survival and 9 immune-related genes related to the prognosis of HCC patients. These cells and genes can be considered as biomarkers for judging the prognosis of HCC, and may serve as important targets for HCC immunotherapy. Highlighting the outcomes of our study, firstly, we screened 3 hub genes to establish an IRGs signature, and verified and evaluated the signature in the multiple data sets, demonstrating the reliability of the signature. Secondly, we have conducted a comprehensive and in-depth study on the correlation between IRGs signature and immune cells and immune checkpoints, which provides a potential direction for the research of HCC immunotherapy. And finally, we established a quantitatively calculated nomogram, which is more conducive to clinical application. However, the limitation of this study is that the public data set lacks some key clinical pathology and neoadjuvant therapy related information of patients. Meanwhile, markers based on immune-related genes need to be applied in clinical patients to verify the predictability of their prognosis.

## Supplementary Information

**Additional file 1:** **Supplementary Figure 1.** The relationship between the abundance ratios of the immune cells and clinical characteristics. (A–C) The relationship between the abundance ratios of each immune cell and stage T, stage and tumor grade. **Supplementary Figure 2.** (A) Volcano plots of the hepatocellular carcinoma gene expression profiles grouping by the risk score. Red/blue symbols classify the upregulated/downregulated genes according to the criteria: |log2FC| > 1.5 and *P*-value < 0.05. (B-E) Represent the enrichment analysis results of genes involved in immune cell infiltration, namely biological processes, cellular components, molecular functions, and KEGG. The main 12 results of each term are shown.

**Additional file 2:** **Supplementary Table 1.** qPCR primer sequence.

**Additional file 3:** **Supplementary Table 2. **Function roles of the 30 hub genes.

## Data Availability

The datasets analysed during the current study are available in TCGA, ICGC and GSE76427. (http://portal.gdc.cancer.gov/, https://dcc.icgc.org/, https://www.ncbi.nlm.nih.gov/geo/query/acc.cgi?acc=GSE76427) All data and outcomes generated during this study are included in this published article and its supplementary information files.

## Abbreviations

HCC: Hepatocellular carcinoma
OS: Overall survival
DEGs: Differential expression genes
TCGA: The cancer genome atlas
ROC: Receiver operating curve
AUC: Area under the curve
IRGs: Immune-related genes
TIME: Tumor immune microenvironment
GO: Gene Ontology
AFP: Alpha-fetoprotein
MHC: Major histocompatibility complex
CTLs: Cytotoxic T lymphocytes
TCM: Memory T cell
TEM: Effector memory T cell
TAMs: Tumor-associated macrophages
