# Supplementary material for: A new nomogram model for prognosis of hepatocellular carcinoma based on novel gene signature that regulates cross-talk between immune and tumor cells
Source: BMC Cancer. 2022 Apr 9;22:379. doi: 10.1186/s12885-022-09465-9 (PMC8994280; doi:10.1186/s12885-022-09465-9)
Supplement: Supplementary file 1 — Additional file 1: Supplementary Figure 1. The relationship between the abundance ratios of the immune cells and clinical characteristics. (A–C) The relationship between the abundance ratios of each immune cell and stage T, stage and tumor grade. Supplementary Figure 2. (A) Volcano plots of the hepatocellular carcinoma gene expression profiles grouping by the risk score. Red/blue symbols classify the upregulated/downregulated genes according to the criteria: |log2FC| > 1.5 and P-value < 0.05. (B-E) Represent the enrichment analysis results of genes involved in immune cell infiltration, namely biological processes, cellular components, molecular functions, and KEGG. The main 12 results of each term are shown. [file 12885_2022_9465_MOESM1_ESM.doc]

| 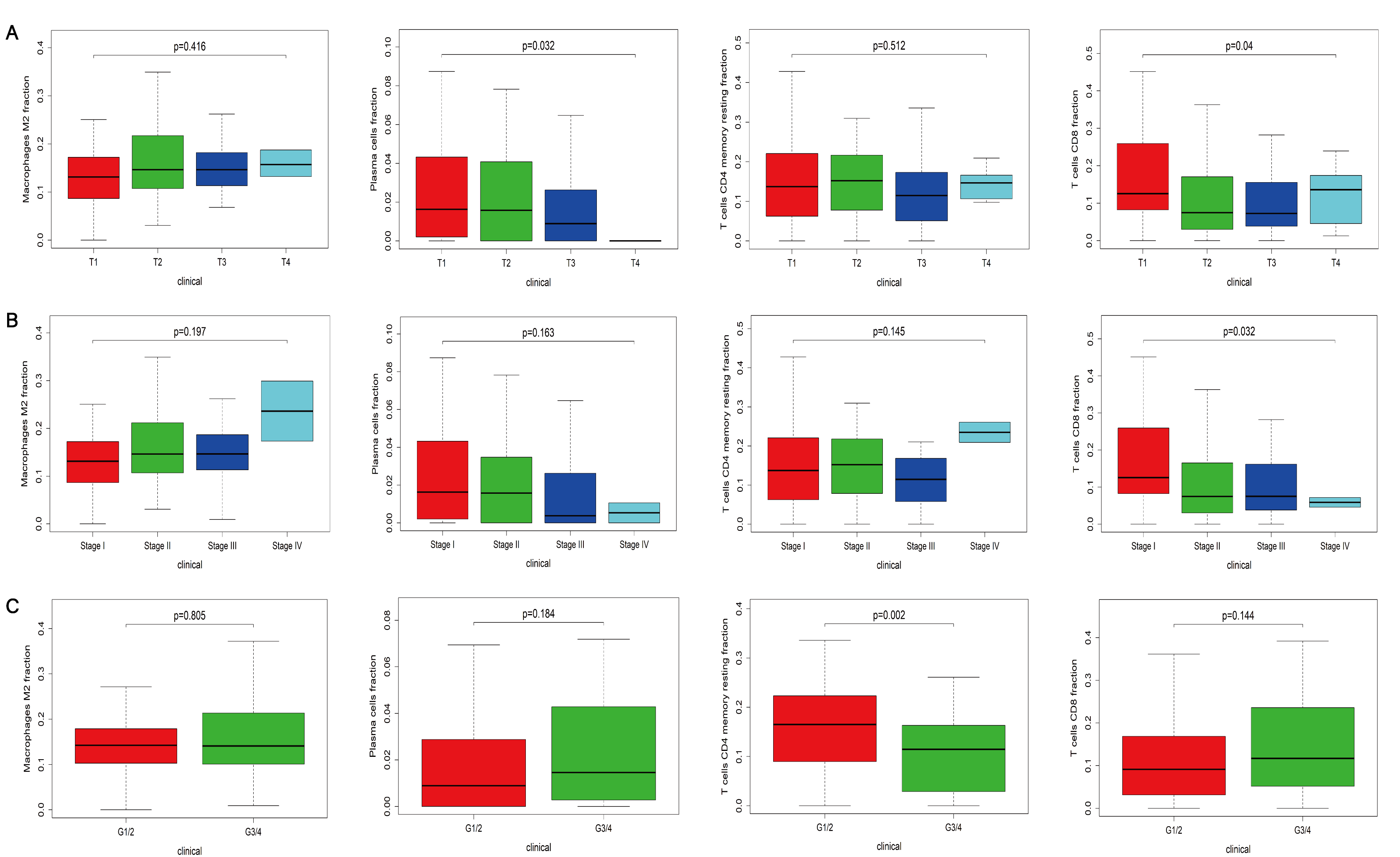 |
| --- |
| **Supplementary Figure 1.** The relationship between the abundance ratios of the immune cells and clinical characteristics. (A–C) The relationship between the abundance ratios of each immune cell and stage T, stage and tumor grade. |
| **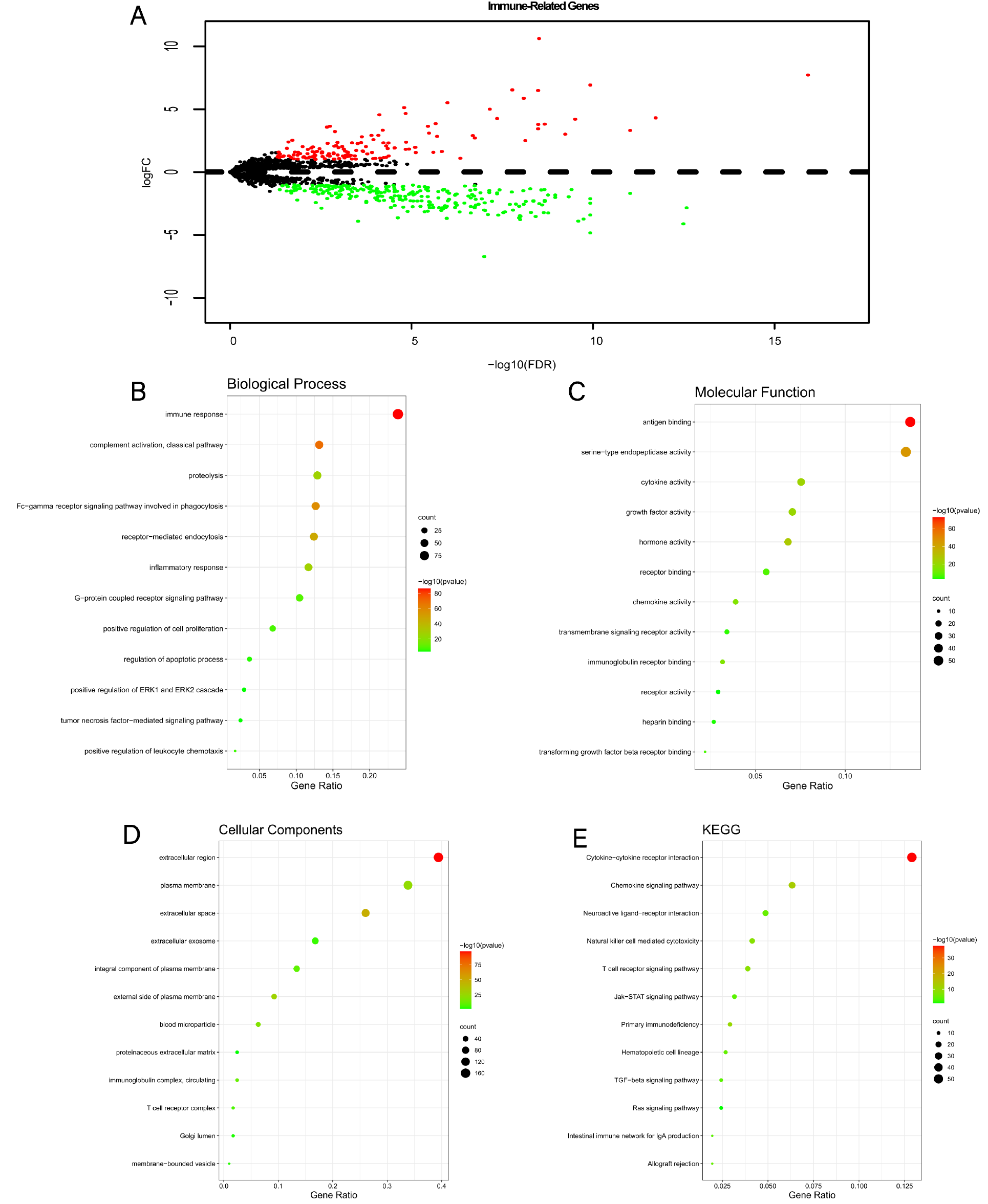** |
| **Supplementary Figure 2.** (A) Volcano plots of the hepatocellular carcinoma gene expression profiles grouping by the risk score. Red/blue symbols classify the upregulated/downregulated genes according to the criteria: |log2FC| > 1.5 and P-value < 0.05. (B-E) Represent the enrichment analysis results of genes involved in immune cell infiltration, namely biological processes, cellular components, molecular functions, and KEGG. The main 12 results of each term are shown. |
