# Supplementary material for: A new nomogram model for prognosis of hepatocellular carcinoma based on novel gene signature that regulates cross-talk between immune and tumor cells
Source: BMC Cancer. 2022 Apr 9;22:379. doi: 10.1186/s12885-022-09465-9 (PMC8994280; doi:10.1186/s12885-022-09465-9)
Supplement: Supplementary file 2 — Additional file 2: Supplementary Table 1. qPCR primer sequence. [file 12885_2022_9465_MOESM2_ESM.docx]

**Supplementary table 1.**

| Gene ID | Species | Primer Sequence |
| --- | --- | --- |
| CD79B | Human | Forward primer: 5’-TGGGGGTGCTATGAGGTGAT-3’ |
|  |  | Reverse primer: 5’-TGCGGGAGAGGAATGATGTTC-3’ |
| S100A9 | Human | Forward primer: 5’-GCTGGTGCGAAAAGATCTGC-3’ |
|  |  | Reverse primer: 5’-GTCACCCTCGTGCATCTTCT-3’ |
| TNFRSF11B | Human | Forward primer: 5’-CCTCTGTGAAAACAGCGTGC-3’ |
|  |  | Reverse primer: 5’-AGGTGTCTTGGTCGCCATTT-3’ |
| CD163 | Human | Forward primer: 5’-CCAGAAGGAACTTGTAGCCACAG-3’ |
|  |  | Reverse primer: 5’-CAGGCACCAAGCGTTTTGAGCT -3’ |
| CD206 | Human | Forward primer: 5’- AGCCAACACCAGCTCCTC -3’ |
|  |  | Reverse primer: 5’- AACGCTCGCGCATTGTC -3’ |
| CD14 | Human | Forward primer: 5’- AAGCACTTCCAGAGCCTGTC -3’ |
|  |  | Reverse primer: 5’- TCGTCCAGCTCACAAGGTTC -3’ |
| CD11b | Human | Forward primer: 5’- CTGTTTACCTGTTTCACGGAAC -3’ |
|  |  | Reverse primer: 5’- GATTGCCTTGACTCTCAGTACT -3’ |
