# Supplementary material for: A new nomogram model for prognosis of hepatocellular carcinoma based on novel gene signature that regulates cross-talk between immune and tumor cells
Source: BMC Cancer. 2022 Apr 9;22:379. doi: 10.1186/s12885-022-09465-9 (PMC8994280; doi:10.1186/s12885-022-09465-9)
Supplement: Supplementary file 3 — Additional file 3: Supplementary Table 2. Function roles of the 30 hub genes. [file 12885_2022_9465_MOESM3_ESM.doc]

| No. | Gene | Full name | Function |
| --- | --- | --- | --- |
| 1 | IL2 | Interleukin 2 | T-cell proliferation and other activities crucial to regulation of the immune response |
| 2 | IL4 | Interleukin 4 | Several B-cell activation processes as well as of other cell types |
| 3 | CCL5 | C-C motif chemokine ligand 5 | Chemoattractant for blood monocytes, memory T-helper cells and eosinophils. |
| 4 | CCR7 | C-C motif chemokine receptor 7 | Receptor for the MIP-3-beta chemokine |
| 5 | CXCL10 | C-X-C motif chemokine ligand 10 | Chemotaxis, differentiation, and activation of peripheral immune cells |
| 6 | MMP9 | Matrix metallopeptidase 9 | Regulate local proteolysis of the extracellular matrix and in leukocyte migration |
| 7 | IFNG | Interferon gamma | Enhance the antiviral and antitumor effects of type I interferon |
| 8 | IL13 | Interleukin 13 | Inhibits inflammatory cytokine production and synergizes with IL2 in regulating interferon-gamma synthesis |
| 9 | CXCL1 | C-X-C motif chemokine ligand 1 | Has chemotactic activity for neutrophils. |
| 10 | SST | Somatostatin | Inhibits the secretion of pituitary hormones |
| 11 | CXCR3 | C-X-C motif chemokine receptor 3 | Mediating the proliferation, survival and angiogenic activity of human mesangial cells |
| 12 | CXCL9 | C-X-C motif chemokine ligand 9 | Chemotactic for activated T-cells |
| 13 | CXCR5 | C-X-C motif chemokine receptor 5 | Involved in B-cell migration into B-cell follicles of spleen and Peyer patches |
| 14 | CD19 | CD19 molecule | Functions as coreceptor for the B-cell antigen receptor complex on B-lymphocytes |
| 15 | CCL19 | C-C motif chemokine ligand 19 | May play an important role in trafficking of T-cells in thymus, and T-cell and B-cell migration to secondary lymphoid organs |
| 16 | CXCL5 | C-X-C motif chemokine ligand 5 | Involved in neutrophil activation |
| 17 | PYY | Peptide YY | Inhibit exocrine pancreatic secretion, have a vasoconstrictory action and inhibitis jejunal and colonic mobility |
| 18 | CXCL13 | C-X-C motif chemokine ligand 13 | Chemotactic for B-lymphocytes |
| 19 | CXCL11 | C-X-C motif chemokine ligand 11 | Chemotactic for interleukin-activated T-cells |
| 20 | IDO1 | Indoleamine 2,3-dioxygenase 1 | Involved in the peripheral immune tolerance |
| 21 | NCR3 | Natural cytotoxicity triggering receptor 3 | Control NK cells cytotoxicity against tumor cells |
| 22 | IL11 | Interleukin 11 | Stimulate the proliferation of hematopoietic stem cells and megakaryocyte progenitor cells and induces megakaryocyte maturation |
| 23 | TNFRSF11B | TNF receptor superfamily member 11b | Inhibits the activation of osteoclasts and promotes osteoclast apoptosis in vitro |
| 24 | ADIPOQ | Adiponectin, C1Q and collagen domain containing | involved in the control of fat metabolism and insulin sensitivity |
| 25 | CD247 | CD247 molecule | Part of the TCR-CD3 complex present on T-lymphocyte cell surface |
| 26 | S100A8 | S100 calcium binding protein A8 | Induce neutrophil chemotaxis and adhesion |
| 27 | PLA2G2A | Phospholipase A2 group IIA | Implications in host antimicrobial defense, inflammatory response and tissue regeneration |
| 28 | LTBP1 | Latent transforming growth factor beta binding protein 1 | Key regulator of transforming growth factor beta |
| 29 | S100A9 | S100 calcium binding protein A9 | Calcium- and zinc-binding protein which induce neutrophil chemotaxis and adhesion |
| 30 | CD79B | CD79b molecule | Initiation of the signal transduction cascade activated by the B-cell antigen receptor complex (BCR) |

**Supplementary Table 2.** Function roles of the 30 hub genes.
